# Evolution of Metabolome and Transcriptome Supports a Hierarchical Organization of Adaptive Traits

**DOI:** 10.1093/gbe/evad098

**Published:** 2023-05-26

**Authors:** Wei-Yun Lai, Kathrin A Otte, Christian Schlötterer

**Affiliations:** Institut für Populationsgenetik, Vetmeduni Vienna, Vienna, Austria; Vienna Graduate School of Population Genetics, Vetmeduni Vienna, Vienna, Austria; Institut für Populationsgenetik, Vetmeduni Vienna, Vienna, Austria; Current affiliation: Aquatic Chemical Ecology, University of Cologne, Cologne, Germany; Institut für Populationsgenetik, Vetmeduni Vienna, Vienna, Austria

## Abstract

Most organismal phenotypes have a polygenic basis, which enables adaptive phenotypic responses on ecological time scales. While adaptive phenotypic changes are highly parallel in replicate populations, this does not apply to the contributing loci. In particular for small populations, the same phenotypic shift can be fueled by different sets of alleles at alternative loci (genetic redundancy). Although this phenomenon is empirically well supported, the molecular basis of the genetic redundancy is not yet understood. To fill this gap, we compared the heterogeneity of the evolutionary transcriptomic and metabolomic response in ten *Drosophila simulans* populations which evolved parallel high-level phenotypic changes in a novel temperature environment but used different allelic combinations of alternative loci. We showed that the metabolome evolved more parallel than the transcriptome, confirming a hierarchical organization of molecular phenotypes. Different sets of genes responded in each evolved population but led to the enrichment of similar biological functions and a consistent metabolic profile. Since even the metabolomic response was still highly heterogeneous across evolved populations, we propose that selection may operate on pathways/networks.

**Significance**

It is now firmly established that polygenic adaptation can be heterogeneous across evolved populations at the genomic level but converges for high-level phenotypes (e.g., fitness). One question towards this is how the heterogeneous genomic responses are transmitted to the parallel changes in high-level phenotypes. To fill this gap, we studied two intermediate molecular phenotypes—transcriptomics and metabolomics. We demonstrated that the consistency of evolutionary response across replicates increases from the transcriptomic level to the metabolomic level, and similar biological functions are enriched by the heterogeneous sets of genes responding in each replicate but lead to consistent metabolic output.

## Introduction

Rapid adaptive response mediated by many loci in ecologically relevant time frames is a hallmark of polygenic adaptation (Sella and Barton 2019). Even large phenotypic changes can be achieved by minor allele frequency changes at many loci (Mackay 2001; Barton and Keightley 2002; Sella and Barton 2019). Nevertheless, the same phenotypic shift can be obtained by several allelic combinations at different genetic loci (Csilléry et al. 2018; Barghi et al. 2019; Therkildsen et al. 2019). This genetic redundancy is particularly important to understand the adaptive response in small to moderately sized populations. Because genetic drift can either act synergistically or antagonistically to selection driven allele frequency changes, genetic redundancy leads to different genomic responses in replicate populations even when the same selection regime is applied to genetically identical populations (Barghi et al. 2020).

This many-to-one relationship, where alleles at many different genes ultimately cause a convergent high-level phenotypic response (typically fitness), has been of interest to both theoreticians and empiricists. While the theory was mostly focused on connecting dynamics of adaptive alleles and phenotypes (Höllinger et al. 2019; Hayward and Sella 2022), empirical work took advantage of pathways and GO categories to characterize polygenic adaptation either based on inconsistent sets of genes or very subtle frequency shifts (e.g., Conte et al. 2012; Daub et al. 2013; Natarajan et al. 2016; Lai and Schlötterer 2022).

**Fig. 1.**
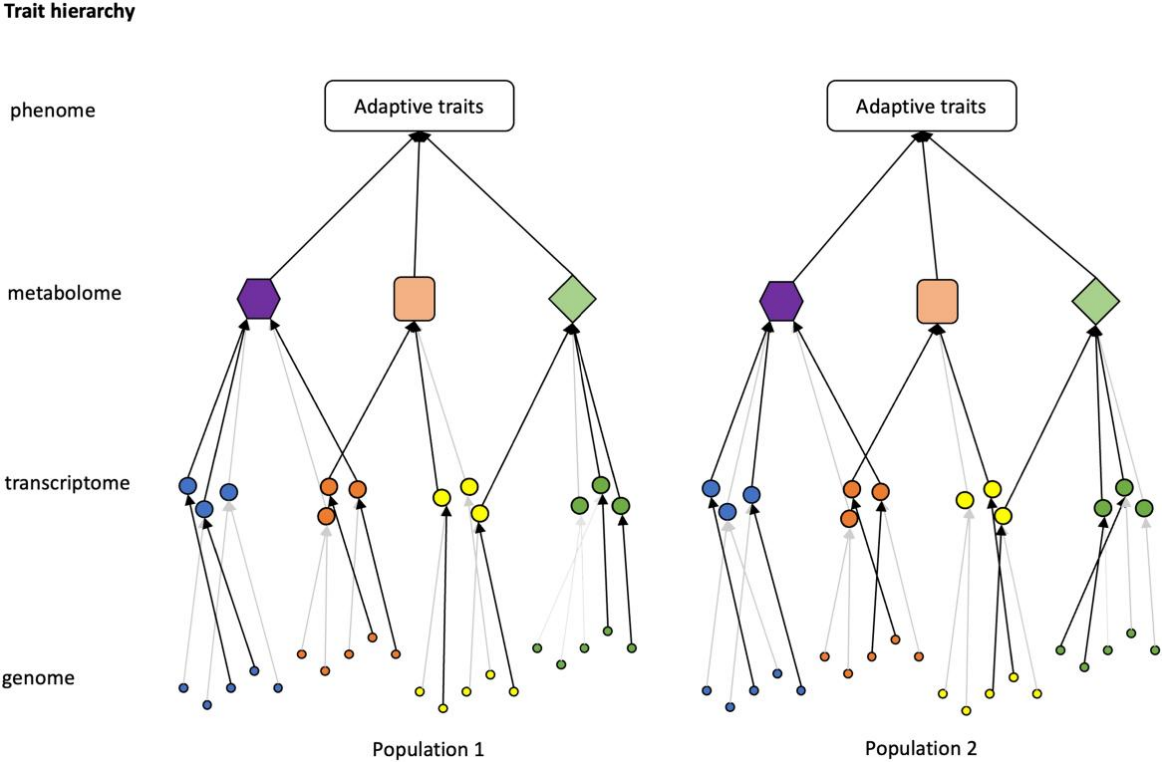
Illustration of trait hierarchy and redundancy in adaptive trait evolution. Traits are hierarchically organized. Different layers indicate different trait hierarchies. An adaptive trait may be mediated by the metabolomic output of several pathways each with different sets of involved genes. Genes with the same colors are assumed functionally redundant. In a population, coding sequence variation and/or regulatory variants in the genome are the raw material for adaptation to any environmental shift. The hierarchical nature of trait organization provides the possibility of redundancy among genomic variants/genes and hence the heterogeneous evolutionary response at genomic/molecular phenotypic levels (arrows indicate all potential adaptive paths across trait hierarchy in the populations: black arrows denote the path taken in each population while gray ones are not taken). Nevertheless, such redundancy and heterogeneity would decline at higher levels such as metabolomes and organismal phenotypes.

Most redundant adaptive variants do not affect fitness directly but rather affect traits organized in a network (Fagny and Austerlitz 2021) or a trait hierarchy (Barghi et al. (2020); figure 1). Seminal work on temperature adaptation of *Escherichia coli* in a highly replicated experimental evolution study uncovered this hierarchy by finding different extent of parallel evolution on different hierarchical levels: while least parallelism was seen for SNPs, the most parallel response was detected for annotated biological pathways (Tenaillon et al. 2012). Very little information is available on how redundant genetic variants are propagated through intermediate phenotypic levels into a high-level parallel phenotypic response. In this study, we aim to explicitly focus on intermediate molecular phenotypes, gene expression, and metabolite abundance, to characterize phenotypic convergence in an experimental system with divergent phenotypic responses that converge on high-level phenotypes.

We take advantage of a recently described experimental evolution study, where ten replicated populations starting from the same founders were adapting independently to a novel temperature regime (18 °C night/28 °C day) over 100 generations (Barghi et al. 2019; Hsu et al. 2020; Jakšić et al. 2020). On the genomic level, heterogeneous responses were observed across the ten evolved populations, but for high-level phenotypes, such as female fecundity, CO_2_ production, and neuronal signaling, a convergent response has been detected (Barghi et al. 2019; Jakšić et al. 2020). We explored whether the concept of trait hierarchies can be applied to gene expression and metabolite abundancies, by asking if gene expression evolution is more redundant than metabolite abundance. We confirm the presence of trait hierarchies by demonstrating more phenotypic convergence for metabolites than for gene expression.

## Results

### Heterogeneity in Gene Expression across Ten Replicated Evolved Populations

**Fig. 2.**
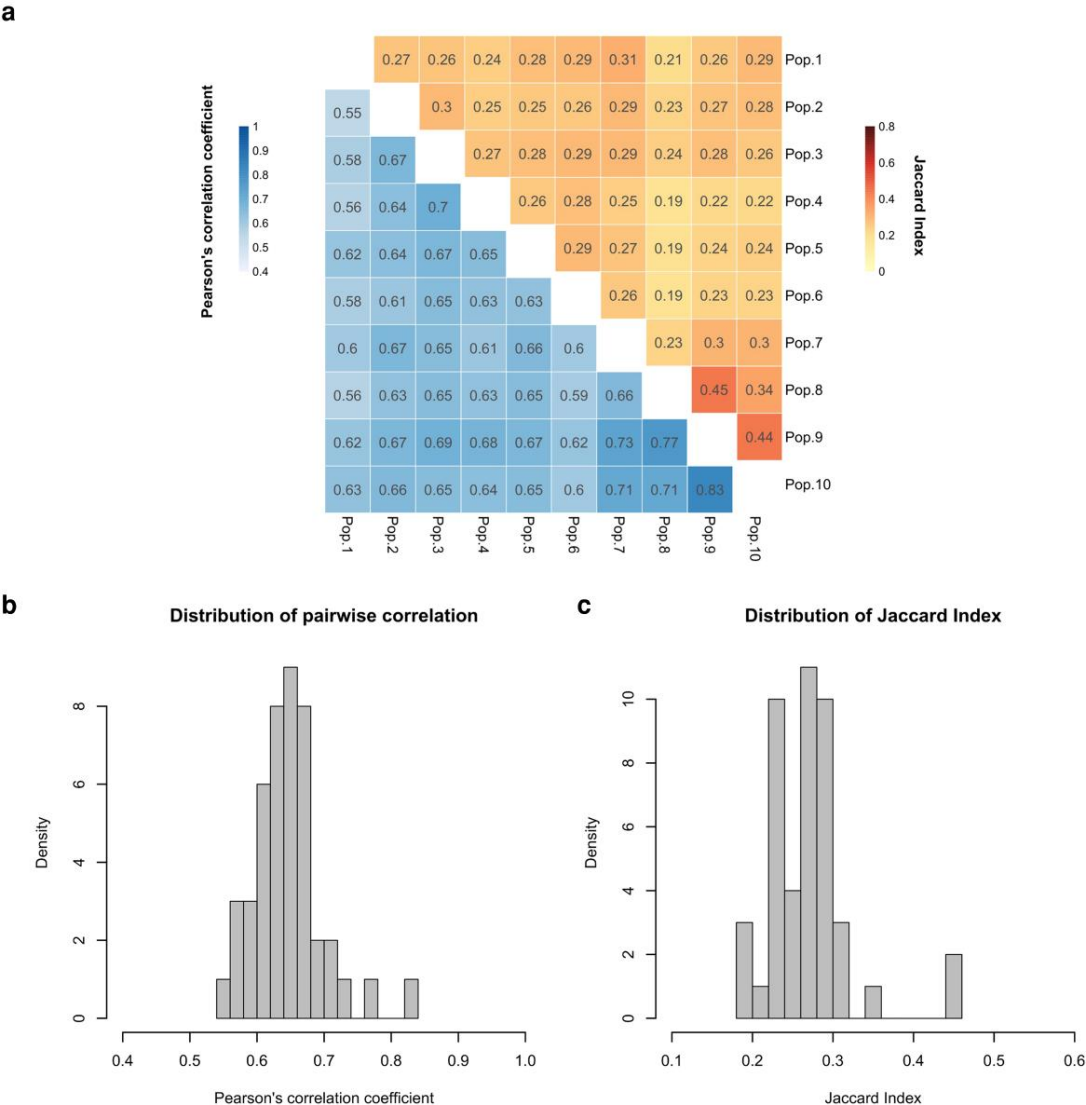
Parallelism of adaptive gene expression changes across ten evolved populations. (*a*) Jaccard similarity indices of pairwise comparisons among differentially expressed genes (orange; upper triangle) and Pearson's correlation coefficients of pairwise comparisons among all genes (blue; lower triangle) in each population. (*b*) Distribution of correlation coefficients for 45 pairwise comparisons between populations from *a* (lower triangle). (*c*) Distribution of Jaccard Indices in 45 comparisons between ten evolved populations from *a* (upper triangle).

Reanalyzing RNA sequencing (RNA-Seq) data from previous studies (Hsu et al. 2020; Jakšić et al. 2020), we investigated gene expression changes in each of the ten *Drosophila* populations which independently evolved for more than 100 generations in a high-temperature (18 °C night/28 °C day) regime. We contrasted three samples from each evolved population which independently experienced the same common garden environment with five reconstituted ancestral population samples, to identify those genes, which evolved a gene expression change. We identified between 904 and 2,423 genes showing significant evolutionary responses in different populations (adjusted *P* < 0.05; supplementary table S1, Supplementary Material online). The similarity of gene expression evolution across populations can be quantified by the Jaccard index of genes with a significant gene expression change in at least one evolved population. Low to intermediate Jaccard similarity indices of all pairwise combination among the ten evolved populations (0.19–0.45) reveal a heterogeneous evolutionary response (fig. 2*a* and *b*). Since the calculation of the Jaccard index depends on a fixed significance cutoff to identify genes with an evolved expression change, we also evaluated the correlation in evolutionary gene expression changes (Log_2_FC; see Materials and Methods) in pairwise comparisons of independently evolved populations. A moderate Pearson's correlation coefficient (0.55–0.83) provided additional evidence of heterogeneous evolutionary response at the gene expression level (fig. 2*c* and *d*). Both measures, Jaccard Index and Pearson's correlation coefficient, were significantly smaller than the null expectation (considering no population-specific effect) (permutation test *P* < 0.01; supplementary fig. S1, Supplementary Material online), suggesting that the observed heterogeneous evolutionary response cannot be explained by stochasticity only. This population-specific signature of gene expression change in combination with the previously described convergence for high-level phenotypes (Barghi et al. 2019) suggests that independently evolved populations may take alternative paths of expression change to mediate the adaptation to the new environment. This indicates that redundancy is not only observed at the level of genomic response (Barghi et al. 2019) but also detected for gene expression changes.

### Heterogeneity in Metabolite Abundance across Six Replicated Evolved Populations

**Fig. 3.**
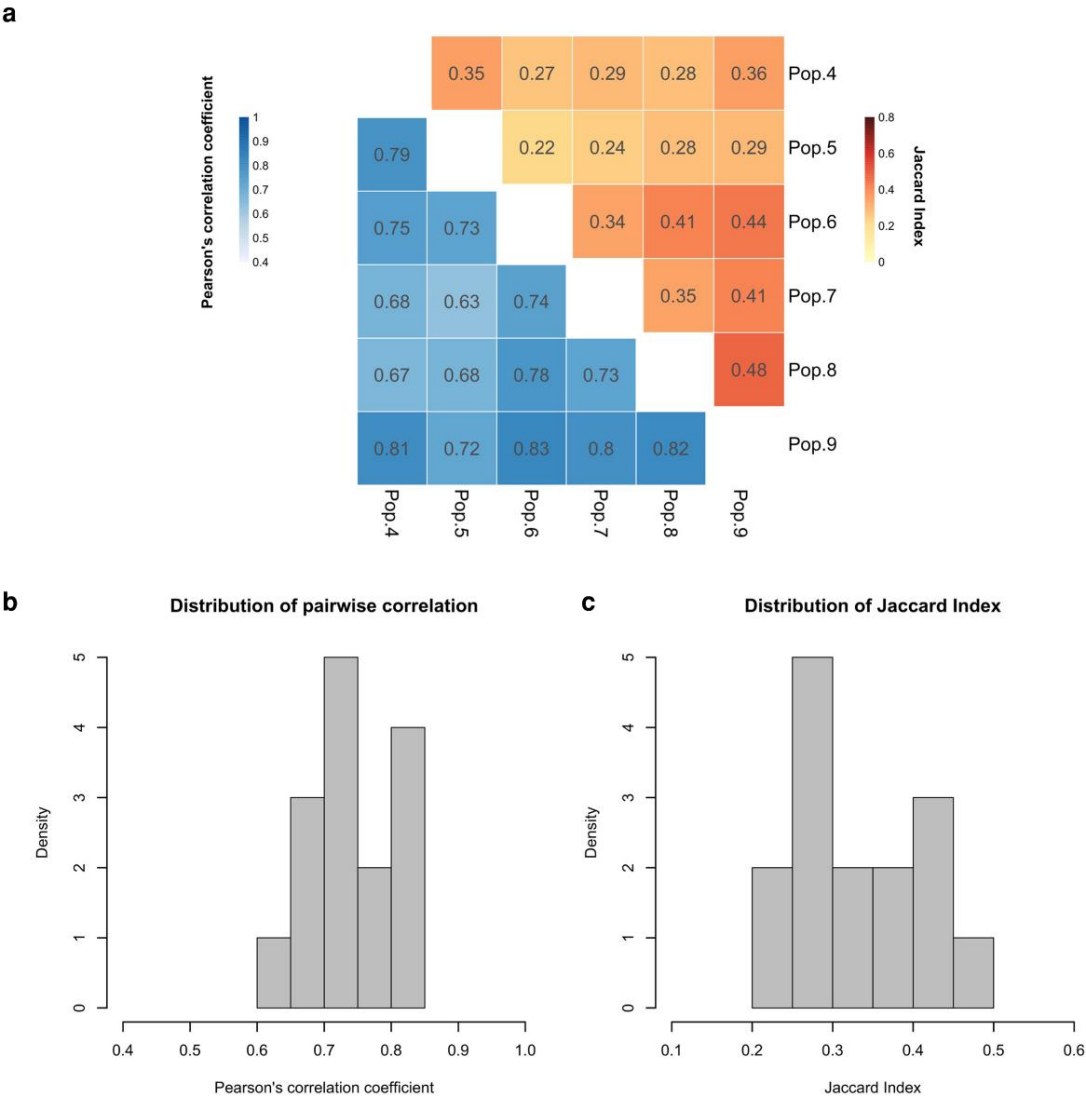
Parallelism of adaptive metabolite abundance change across six evolved populations. (*a*) Jaccard similarity indices of pairwise comparisons between the DE metabolites identified in each population (orange; upper triangle) and Pearson's correlation coefficients of pairwise comparisons among all metabolites (blue; lower triangle). (*b*) Distribution of correlation coefficient of 15 comparisons among populations from *a* (lower triangle). (*c*) Distribution of Jaccard indices of 15 pairwise DE metabolite comparison between six populations from *a* (upper triangle).

Metabolites are the precursors, intermediate, or end products in enzymatic pathways, and their abundance is the outcome of the activities of gene products (Fiehn 2002). Using samples from the same common garden experiment that was used for the RNA-Seq analysis, we investigated the metabolome of five reconstituted ancestral and six randomly chosen evolved populations (each with three samples) using high-performance liquid chromatography and mass spectrum (HPLC-MS). In total, we identified and quantified 940 compounds (supplementary table S2, Supplementary Material online). Contrasting three samples from each evolved population to five reconstituted ancestral population samples, we identified metabolites with significant differences in metabolite abundance in each evolved population. 133 to 162 metabolites per evolved population changed significantly in abundance after 100 generations of adaptation to a novel environment (adjusted *P* < 0.05; supplementary table S2, Supplementary Material online). The pairwise Jaccard similarity indices and Pearson's correlation coefficients were not high (Jaccard similarity indices [0.22–0.48] and Pearson's correlation coefficient [0.62–0.82]), indicating heterogeneity for metabolites (fig. 3). Importantly, both the Jaccard similarity index and the correlation coefficient were significantly smaller than the null expectation of no population-specific evolutionary effects (permutation test [*P* < 0.05, supplementary fig. S2, Supplementary Material online]), evidencing a significant level of heterogeneity between evolved populations at the metabolomic level as well. However, the heterogeneity of the transcriptome is considerably more pronounced than for the metabolome (supplementary figs. S1 and S2, Supplementary Material online).

### Heterogeneous Gene Expression Evolution Is Translated into Consistent Metabolite Evolution

**Fig. 4.**
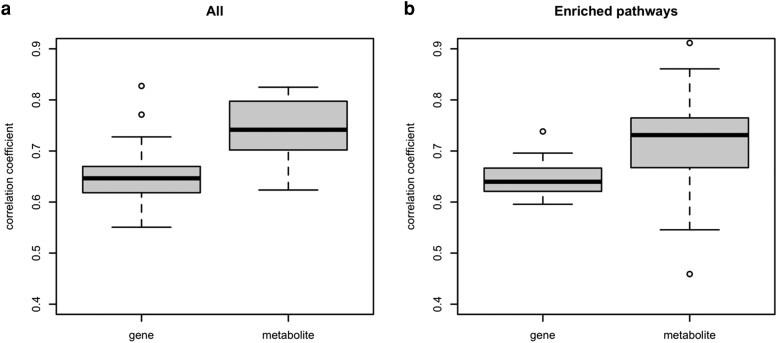
Similarity of the evolutionary response in ten evolved populations for gene expression and metabolite abundance. (*a*) Pairwise log_2_FC correlation coefficient between six populations (pop4, pop5, pop6, pop7, pop8, and pop9) for all expressed genes (*n* = 10,780) and metabolites (*n* = 940). A significantly higher similarity is observed at the metabolite level (*t*-test, *P* < 0.001). (*b*) Pairwise log_2_FC correlation coefficients between six populations are shown for the genes (*n* = 239) and metabolites (*n* = 48) from pathways that are enriched in all six evolved populations. Metabolites were more similar between populations than gene expression (*t*-test, *P* < 0.05).

Metabolites are regulated by complex interactions at the level of the genome and transcriptome and are considered closest to the organismal (high-level) phenotype (Zhou et al. 2020). Given the convergent evolution of high-level phenotypes of the focal populations (Barghi et al. 2019), we were interested whether the evolution of the metabolome is more consistent across evolved populations than the transcriptome. Pairwise Pearson's correlation coefficients of evolved changes in gene expression (log_2_FC) between evolved populations were significantly higher for metabolome based on six evolved populations (pop4, pop5, pop6, pop7, pop8, and pop9) than the transcriptome (*t*-test, *P* < 0.001; see Materials and Methods) (fig. 4*a*). The same significant difference was observed for the Jaccard index, which is based on genes with significant changes in the evolved populations (supplementary fig. S3*a*, Supplementary Material online). To rule out that a larger number of observations for gene expression affected the results, we randomly sampled the same number of expressed genes as the number of tested metabolites for calculating the Jaccard Index and Pearson's correlation coefficient (see Materials and Methods). The results remained unaffected by the downsampling process (Supplementary fig. 4*a* and *b*).

To characterize the pathways that are affected both transcriptionally and metabolically during thermal adaptation, we performed a joint pathway analysis which integrates the information from both gene expression and metabolites (see Materials and Methods). We identified between 49 and 83 significantly enriched KEGG pathways in each evolved population, 29 of them were shared across all six evolved populations for which both metabolomics and transcriptomic data were available (table 1 and supplementary fig. S5, Supplementary Material online). These enriched pathways are related to the sugar, lipid metabolism, synthesis of neural transmitter precursors, and energy metabolism (table 1 and supplementary table S3, Supplementary Material online).

**Table 1:** Significantly Enriched Pathways in All Six Evolved Populations

KEGG ID	KEGG pathway	FDR value^a^	Pathway category
00020	Citrate cycle (TCA cycle)	0.031	Carbohydrate metabolism
00051	Fructose and mannose metabolism	0.033	
00052	Galactose metabolism	0.033	
00500	Starch and sucrose metabolism	0.043	
00520	Amino sugar and nucleotide sugar metabolism	0.024	
00190	Oxidative phosphorylation	0.021	Energy metabolism
00561	Glycerophospholipid metabolism	0.021	Lipid metabolism
00600	Sphingolipid metabolism	0.021	
00230	Purine metabolism	0.031	Nucleotide metabolism
00240	Pyrimidine metabolism	0.031	
00270	Cysteine and methionine metabolism	0.021	Amino acid metabolism
00290	Valine, leucine, and isoleucine biosynthesis	0.021	
00330	Arginine and proline metabolism	0.043	
00350	Tyrosine metabolism	0.021	
00360	Phenylalanine metabolism	0.021	
00400	Phenylalanine, tyrosine, and tryptophan biosynthesis	0.029	
00450	Selenocompound metabolism	0.021	
00130	Ubiquinone and other terpenoid-quinone biosynthesis	0.024	Metabolism of cofactors and vitamins
00740	Riboflavin metabolism	0.024	
00790	Folate biosynthesis	0.021	
00903	Limonene and pinene degradation	0.021	Metabolism of terpenoids and polyketides
00980	Drug metabolism—cytochrome 450	0.021	Xenobiotics biodegradation and metabolism
00982	Metabolism of xenobiotics by cytochrome P450	0.024	
04142	Lysosome	0.021	Transport and catabolism
04145	Phagosome	0.021	
04080	Neuroactive ligand-receptor interaction	0.024	Signaling molecules and interaction

a The mean FDR values among six populations are reported.

To demonstrate that the heterogeneous response in gene expression evolution can result in similar changes in metabolite abundance in the potentially selected pathways, we focused our subsequent analysis on pathways that are consistently detected across all six populations and have at least five annotated metabolites. We compared the correlation of evolved changes in gene expression and changes metabolite abundance that are annotated in these pathways. Consistent with the full data set, we found that both correlation coefficients and Jaccard Indices were significantly lower for gene expression than for metabolites (*t*-test, *P* < 0.05 for correlation coefficient (fig. 4*b*); *t*-test, *P* < 3.26*e*^−06^ for the Jaccard Index (Supplementary fig. 3*b*)). This observation suggests that the heterogeneity at the gene expression level is transmitted to similar metabolomic profiles through shared biological functions or features across evolved populations.

## Discussion

This study benefitted from a common garden experiment, which provided sufficient samples to enable the comparison of evolutionary response of replicated populations for the transcriptome and metabolome from the same experiment. In the same common garden experiment of this population, a convergent evolutionary response has been seen for high-level phenotypes (e.g., fecundity, metabolic rate, and neuronal signaling (Barghi et al. 2019; Hsu et al. 2020; Jakšić et al. 2020)). To understand how heterogeneous genomic responses converge to similar changes of organismal phenotypes, we measured the evolutionary heterogeneity at two intermediate molecular levels—transcriptome and metabolome. One interesting observation in this study was the high level of heterogeneity among the evolved populations at both levels. This implicates the presence of redundancy and alternative molecular paths to achieve organismal adaptation.

We expected more consistent signals across evolved populations for the metabolome than for the transcriptome, reasoning different hierarchy levels for these two classes of molecular phenotypes (fig. 1). This expectation is consistent with the results of Zhou et al. (2020). The authors built a network incorporating polymorphic markers, transcriptome, and metabolome that are associated with the variation in higher organismal traits. This integrated network suggested that metabolites are regulated by complex interactions of genomic variation and transcriptomic differences. Consistent with these expectations, we found that the evolutionary response was more parallel for the metabolome than for the transcriptome not only on a genome-wide scale (fig. 4*a* and Supplementary fig. 3*a*) but also for putative adaptive pathways that are consistently enriched in six evolved populations (fig. 4*b* and Supplementary fig. 3*b*). Heterogeneous sets of genes of the same pathway may respond to selection in different evolved populations, but a similar metabolic output is reached. For example, in the purine metabolism, some of the enzyme-coding genes with heterogeneous expression changes across evolved populations are connected to metabolites that show more parallel changes among populations (e.g., GMP, guanosine, deoxyguanosine, and their connected enzyme-coding genes) (supplementary fig. S6, Supplementary Material online). This higher level of convergence for the metabolome than transcriptome may result from the hierarchical organization of the two phenotype classes as previously proposed by Zhou et al. (2020).

Although consistent with a hierarchical organization of traits, we caution that different levels of convergence between the metabolome and transcriptome may have other causes. First, metabolomics and transcriptomics may have different levels of measurement errors. Larger measurement errors can lower the correlation of the evolutionary response across populations. We observed significantly larger measurement errors for metabolite abundance (supplementary fig. S7, Supplementary Material online), suggesting that we underestimated the convergence for the metabolome. Because we found more convergence at the metabolomic level than for the transcriptome, the different levels of technical noise for the two methods are conservative and do not affect our conclusion. Second, the identification of metabolites and transcripts differs. While we used untargeted metabolomics, the identification of metabolites is limited by the available reference metabolites. Transcriptomic analysis, on the other hand, relies on the quality of the annotation. Furthermore, the annotation of all reaction steps in various of biological pathways with involved genes and metabolites is even more challenging. Third, the relationship between genes and metabolites in a pathway is most likely much more complex than the linear relationship assumed in this study. Rather, complex network structures with feedback loops, substrates competition, etc. are biologically more realistic (Sontag et al. 2004; Lempp et al. 2019). Lastly, we studied pools of individuals, which average across heterogeneities among individuals and tissues. It is possible that accounting for these heterogeneities may uncover different trends than those seen in this study, but the scale of such experiments is out of scope for this study. We anticipate this study to spearhead future investigations when more mature and cost-effective techniques come into place.

Here, we present not only a systematic study on the molecular mechanisms underlying phenotypic adaptation, but we also identified the pathways that evolved in response to the hot environment. Many of them have been associated with important roles in temperature adaptation. For example, the regulation of purine metabolism has been reported to reduce the heat-induced oxidative stress (Tian et al. 2022) and is involved in the adaptation to high temperature in several species (Paget et al. 2014; Chen et al. 2021; Jahan et al. 2022). The pentose phosphate pathway contributes to thermal adaptation in *Thermus filiformis*: under heat conditions, glucose was predominantly metabolized via the pentose phosphate pathway instead of the glycolysis pathway (Mandelli et al. 2017). The lysosome was also found to be involved in adaptation and tolerance to temperature stress in various organisms (Camus et al. 2000; Wang et al. 2018). Furthermore, several of the enriched pathways can be connected to previously identified selected traits in this experimental evolution study using a polymorphic *D. simulans* population from Florida. Tyrosine metabolism and phenylalanine metabolism are the pathways related to the synthesis of dopamine, one important neural transmitter. Evolution in dopaminergic neuronal activities is one of the evolved phenotypes documented for the populations (Jakšić et al. 2020). Neuroactive ligand–receptor interaction, a pathway related to signaling molecules and interaction, is also identified in all six populations. Furthermore, several evolved metabolites within the enriched pathways may be involved in temperature adaptation (table 2). For example, guanosine diphosphate (GDP)-mannose (consistently detected in all six evolved populations) is part of the amino sugar and nucleotide sugar metabolism has been reported to be involved in the acclimatization to heat-induced abiotic stress in several species (Bizri et al. 1984; Hoeberichts et al. 2008) as well as thermal adaptation (Gu and Hilser 2009). Deoxyguanosine, which is significantly changing in all six evolved populations, participates in oxidative damage-related processes (Greenberg 2004). Further functional validation experiments on these metabolites may help us to better understand the molecular mechanism underlying temperature adaptation.

**Table 2:** Metabolites That Are Significantly Evolved in at Least 3 Evolved Populations

Metabolite	KEGG ID	Belonging pathway	$\bar{X_{\mathrm{evo}}}-\bar{X_{\mathrm{anc}}}$ ^a^
GDP-mannose	C00096	Amino sugar and nucleotide sugar metabolism; fructose and mannose metabolism	−0.278
Deoxyguanosine	C00330	Purine metabolism	0.235
Orotate	C00295	Pyrimidine metabolism	0.211
Uridine	C00299	Pyrimidine metabolism	−0.326
D-Glucosamine	C00329	Amino sugar and nucleotide sugar metabolism	−0.297
n-Acetylputrescine	C02714	Arginine and proline metabolism	−0.300
Guanosine	C00387	Purine metabolism	0.222
FAICAR	C04734	Purine metabolism	0.316

a $\bar{X_{\mathrm{evo}}}-\bar{X_{\mathrm{anc}}}\;$ indicates the difference in abundance between the average of evolved samples (*n* = 18) and ancestral samples (*n* = 5).

One important challenge for understanding adaptation is the identification and characterization of an adaptive trait. Genetic redundancy in combination with trait hierarchies provides the potential to characterize selected phenotypes: heterogeneous responses across evolved populations indicate that these phenotypes cannot represent the direct target of selection, for which a convergent response across evolved populations is expected (Barghi et al. 2020). Thus, in theory, the phenotype at the lowest hierarchical level which shows convergent adaptive response is a good candidate for the selected traits. Higher trait levels integrate too many subphenotypes to identify the actual selection target. A good example is fitness, which will converge, but is determined by so many traits that a convergent increase in fitness does not allow many conclusions about the selected phenotype(s). Here, our results show to what extent molecular phenotypes allow for a better characterization of the selected trait. Transcriptomic and metabolomic data offer the advantage of high-throughput analyses, but we also demonstrated the limitations of theses phenotypes to identify the selected phenotypes. Our analysis of some putatively selected pathways misses many metabolites. This may reflect the unbiased nontargeted metabolomics approach and the limited reference metabolite annotation. Targeted metabolomic profiling for all metabolites of a pathway, if possible, could be complementary. More refined expression and metabolomics profiling with single-cell analysis may provide reliable molecular networks and help characterizing a more resolved picture of the responses to selection. Nevertheless, it is not yet clear whether selection targets can be broken down to individual molecular entities, such as a metabolite. One approach to address this question empirically is to perform experimental evolution with a well-defined selection target and explore the ability to identify it from molecular phenotypes. Furthermore, crosses between evolved populations may also be a promising approach for future work.

## Materials and Methods

### Experimental Evolution and Common Garden Experiment

The experimental evolution and common garden experiments are described in Barghi et al. (2019); Hsu et al. (2020); Jakšić et al. (2020). Briefly, ten outbred populations seeded from 202 isofemale lines were exposed to a laboratory experiment at 28/18 °C with 12 hr light/12 hr dark photoperiod for more than 100 generations. Each evolving population consisted of 1,000 to 1,250 adults at each generation. The collection of samples from the evolution experiment for RNA-Seq and metabolite profiles was preceded by two generations of common garden. The common garden experiment (CGE) was performed at generation 103 in the hot environment (Barghi et al. 2019; Hsu et al. 2020; Jakšić et al. 2020). In brief, an ancestral population was reconstituted by pooling five mated females from 184 founder iso-female lines. No significant allele frequency differences are expected between the reconstituted ancestral populations and the original ancestral populations initiating the experiment (Nouhaud et al. 2016). Furthermore, we do not anticipate that deleterious alleles acquired during the maintenance of the iso-female lines had a major impact on the phenotypic variance in the reconstituted ancestral population. The reason is that novel deleterious mutations occurring during the maintenance of the iso-female lines are present in a single iso-female line only. Given the large number of iso-female lines (184), such deleterious alleles occur in a low frequency in the reconstituted population with a small influence on the phenotypic variance (Walsh and Lynch 2018). Furthermore, most of these deleterious alleles are present in heterozygous individuals and masked because deleterious alleles tend to be recessive (Charlesworth and Charlesworth 2010).

Five replicates of the reconstituted ancestral population and ten independently evolved populations at generation 103 were reared for two generations with egg-density control (400 eggs/bottle) at the same temperature regime as in the evolution experiment. Each independently evolved population has three biological replicates of 50 5-day-old adult males, which were kept separately for the two generations of common garden. Five biological replicates from the ancestral populations and three biological replicates from all evolved populations (pop1, pop2, …, pop10) were subjected to RNA Sequencing. For a more detailed description of the RNA extraction and library preparation, see Hsu et al. (2020); Jakšić et al. (2020).

### Metabolomic Profiling

For an unbiased metabolomic profiling, we did not focus on an a priori determined set of metabolites but rather used an untargeted approach. We used samples from the same CGE (Barghi et al. 2019; Hsu et al. 2020; Jakšić et al. 2020), to quantify metabolites from the reconstituted ancestral population with five biological replicates and six randomly picked, independently evolved populations (pop4, pop5, pop6, pop7, pop8, and pop9) with three biological replicates each. Samples of 50 male flies from each biological replicate were used for metabolite extraction. Metabolite extraction was performed in MeOH:ACN:H20 (2:2:1 *v*/*v*) using a bead mill, followed by one freezing/thawing cycle and a sonication step. Homogenized samples were then put at −20 °C for 1 h for the proteins to precipitate. After protein removal, samples were dried in a speedvac and reconstituted in ACN:H2O 1:1 (*v*/*v*). All samples were stored at −80 °C until measurement. Metabolomic measurements were performed by Vienna Bio-Center (VBC). Samples were diluted 1:1 with 80% ACN, assigned randomly into the autosampler, and measured randomly in full MS mode on a ZIC-pHILIC. A blank containing the dilution solvent and a pooled FlyQC (5 *µ*l of each sample) were measured before and after 6 samples and used for background correction and normalization, respectively. QC samples were also measured in discovery and confirmation mode to obtain additional MS2 spectra for identification. Raw data were extracted and passed through quality control processes. Peak identification was performed using Compound Disocoverer 3.1 based on the annotation in mzCloud database.

### RNA-Seq Data Analysis

RNA-Seq data were retrieved from European Nucleotide Archive (ENA) with the study accession number PREJEB35504 and PRJEB35506 and reanalyzed following the standard analytical pipeline (Hsu et al. 2020; Jakšić et al. 2020). Only genes with at least 0.1 normalized counts per million bases (CPM) across all samples were considered expressed for further analysis. Because we were interested in the evolutionary response of each independent evolution population, we therefore contrasted the three biological samples from each evolved population to the five biological samples from the ancestral population. The differential expression (DE) analysis was done separately in each of the ten evolved populations. We utilized the generalized linear modeling function implemented in edgeR (Robinson et al. 2010) to fit the expression to the model (y=evo+ε) in which *y* stands for the gene expression, evo is the effect of evolution, and ε is the random error. A likelihood ratio test was performed to test the effect of evolution. *P* value adjustment was performed using the Benjamini–Hochberg false discovery rate (FDR) correction (Benjamini and Hochberg 1995). After identifying DE genes (adjusted *P* < 0.05) in each population, we calculated Jaccard similarity indices (Real and Vargas 1996) of the DE gene sets for all pairwise combinations of the ten evolved populations (45 combinations in total). Jaccard index was calculated as follows:


$$JI=\frac{n\;(A\cap B)}{n\;(A\cup B)},$$

where *A* and *B* referred to the DE gene sets of any two evolved populations. The Jaccard index ranges from 0 to 1. Higher values indicated higher consistency between any pair-wise combination of evolved populations.

For a second measure for similarity between evolved populations based on the quantitative estimates for gene expression in our analysis, we calculated Pearson's correlation coefficient for the expression changes (log_2_FC) of all genes (*n* = 10,780) for all pairwise combinations of evolved populations. The log_2_FC was calculated as follows:


$$\mathrm{lo}g_{2}\mathrm{FC}=\mathrm{lo}g_{2}\left(\frac{\bar{y_{\mathrm{evo}}}}{\bar{y_{\mathrm{anc}}}}\right),$$

where $\bar{y_{\mathrm{evo}}}$ is the mean expression value (CPM) per gene across three evolved samples and $\bar{y_{\mathrm{anc}}}\;$ is the overall mean expression value (CPM) per gene across five ancestral samples.

Finally, we applied a permutation test to investigate whether the observed Jaccard Index/Pearson's correlation coefficient detected in the evolved populations could be caused by random processes (i.e., experimental noise or the sampling process) (Anderson 2001). We randomly permuted the expression values per gene across all 30 biological samples from the evolved populations. With the permuted expression values, we compared them to the five ancestral samples to reidentify the DE genes/recalculate the log_2_FC for all genes and calculated the pairwise Jaccard Index/Pearson's correlation coefficient in each evolved population under null expectation considering no population-specific effects. The procedure was repeated 100 times to generate a null distribution considering no population-specific effects. Our empirical observations were then tested against the null distribution. A smaller value indicates higher heterogeneity (i.e., more deviation from random expectation). The *P* value was calculated as the proportion of sampled permutations where the mean Jaccard index/Pearson's correlation coefficient was smaller than the observed mean. We considered *P* < 0.05 as an indication of a heterogeneous evolutionary response across evolved populations.

### Metabolomic Data Analysis

The normalized area for each detected compound in each sample was log transformed for subsequent analysis. In total, 940 metabolites were detected in all samples (log-transformed normalized area > 10). For the identification of differentially expressed metabolites during adaptation in each evolved population, we applied a permutation test to compare the abundance of each metabolite for five reconstituted ancestral samples to three biological samples for a given evolved population. For each metabolite, we randomly reassigned eight observations into ancestral (*n* = 5) and evolved (*n* = 3) groups and the difference in mean between two groups were calculated. The procedure was repeated for 100 times to generate a null distribution. Observation deviating on both sides of the null distribution indicate significant differences in abundance between the ancestral and evolved samples. Hence, the *P* value for each metabolite of the test is calculated as the proportion of sampled permutations where the absolute difference was greater than the observed absolute difference. *P* value adjustment was performed using Benjamini–Hochberg FDR correction. Metabolites with adjusted *P* < 0.05 are considered significant. The procedure was done separately in each evolved population.

Like the RNA-Seq analysis (see above), we calculated the Jaccard index and Pearson's correlation coefficient for all pairwise combinations of the six evolution populations (15 combinations in total). For Jaccard index calculations, we used the significantly evolved metabolites in each evolved population while for Pearson's correlation coefficients, we used the evolutionary abundance changes (log_2_FC) of all metabolites (*n* = 940) in each evolution populations.

In correspondence to the permutation procedure described for the RNA-Seq data, we also applied the same approach to the metabolome. Here, we permuted the metabolite abundances across all 18 biological samples belonging to the six evolved populations.

### Comparison of the Consistency between Gene Expression and Metabolite

The distribution of Jaccard indices/Pearson's correlation coefficients for changes in gene expression and metabolite abundance was compared to understand the consistency between these two molecular phenotypes in their evolution response using *t*-test. We restricted the comparison of those six evolved populations (pop4, pop5, pop6, pop7, pop8, and pop9) for which transcriptomic and metabolomic data were available. To account for the different number of total genes/metabolites, we downsampled the number of expressed genes (*n* = 10,780) to the number of total metabolites (*n* = 940) and calculated the pairwise correlation coefficient in log_2_FC based on 940 genes. This was repeated 100 times, and the mean distribution was compared to the mean observation of metabolites. For the comparison of Jaccard indices, we downsampled the number of significant DE genes into the number of significant metabolites in each evolved population and calculated the Jaccard indices based on the downsampled gene set. This procedure was repeated for 100 times, and the mean distribution was compared to the mean observation of metabolites.

### Joint Pathway Analysis

We performed joint KEGG pathway analysis to integrate pathway-level analysis of transcriptomics and metabolomics data. For each evolution population, we performed quantitative enrichment analysis on transcriptomic and metabolomic data using R packages “globaltest” and “metaboanalystR” (Chong and Xia 2018; Goeman et al. 2022). Briefly, quantitative enrichment analysis is based on the *globaltest* algorithm (Chong and Xia 2018; Goeman et al. 2022). A list of log_2_FC across all genes/metabolite is provided and does not require a list of significantly changed genes/metabolites. The raw *P* values were integrated with Fisher's method *fisher.method()* (R package “metaseqR”) (Fisher 1925; Moreau et al. 2003). Benjamini–Hochberg FDR correction was performed on the integrated *P* value. Pathways with adjusted *P* < 0.05 are considered significant. *Pathview* (Luo et al. 2017) was used for the pathway visualization of both gene expression and metabolomic data.

## Supplementary Material

evad098_Supplementary_Data

## Data Availability

All sequencing data are available in European Nucleotide Archive (ENA) under the accession numbers PRJEB35504, PRJEB35506, and PRJEB37011. Metabolomic data are available at the MetabiLights database (ID: MTBLS7882) and at https://github.com/cloudweather34/metabolome.
