# Supplementary material for: Evolution of Metabolome and Transcriptome Supports a Hierarchical Organization of Adaptive Traits
Source: Genome Biol Evol. 2023 May 26;15(6):evad098. doi: 10.1093/gbe/evad098 (PMC10246829; doi:10.1093/gbe/evad098)
Supplement: evad098_Supplementary_Data [file evad098_supplementary_data.zip › Supplementary_information_v1.docx]

**
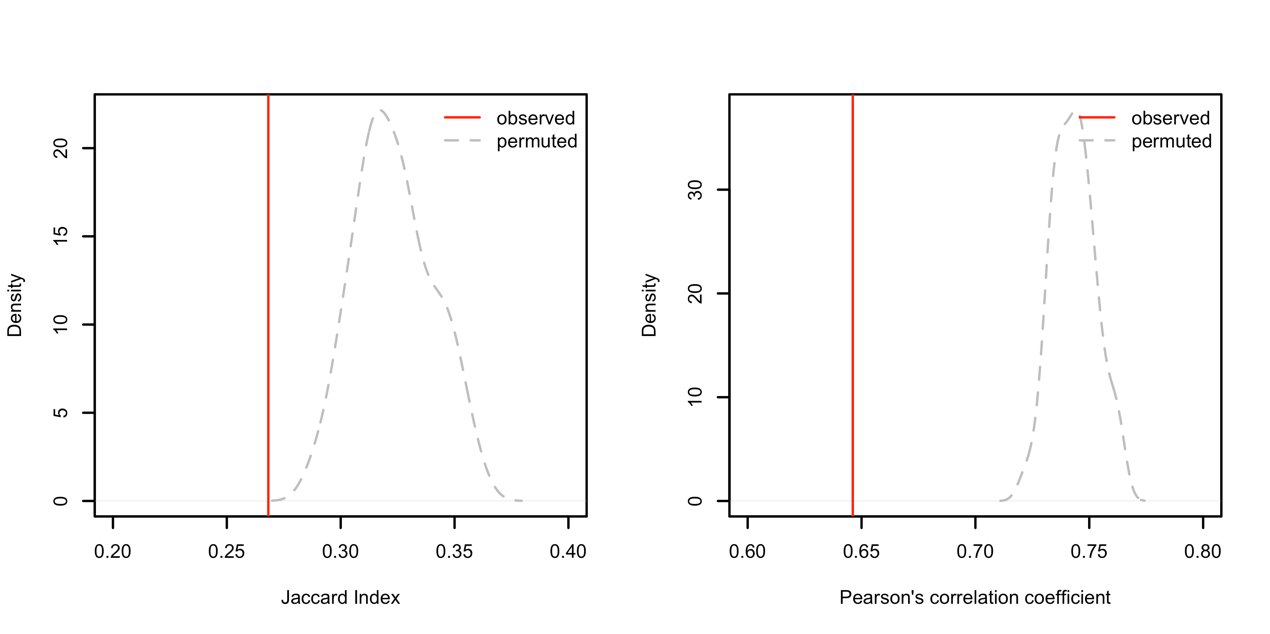
**

a

b

**Supplementary figure 1. Mean pair-wise a. Jaccard Index/ b. Pearson’s correlation coefficient for transcriptome data.** The observed mean pairwise Jaccard Index/Pearson’s correlation coefficient (red) is significantly different from the null expectation considering no population-specific evolutionary effect (grey; 100 times of permutation) (p-value = 0 for both Jaccard Index and r). These results indicated that the observed heterogeneity cannot be solely explained by stochasticity.

**
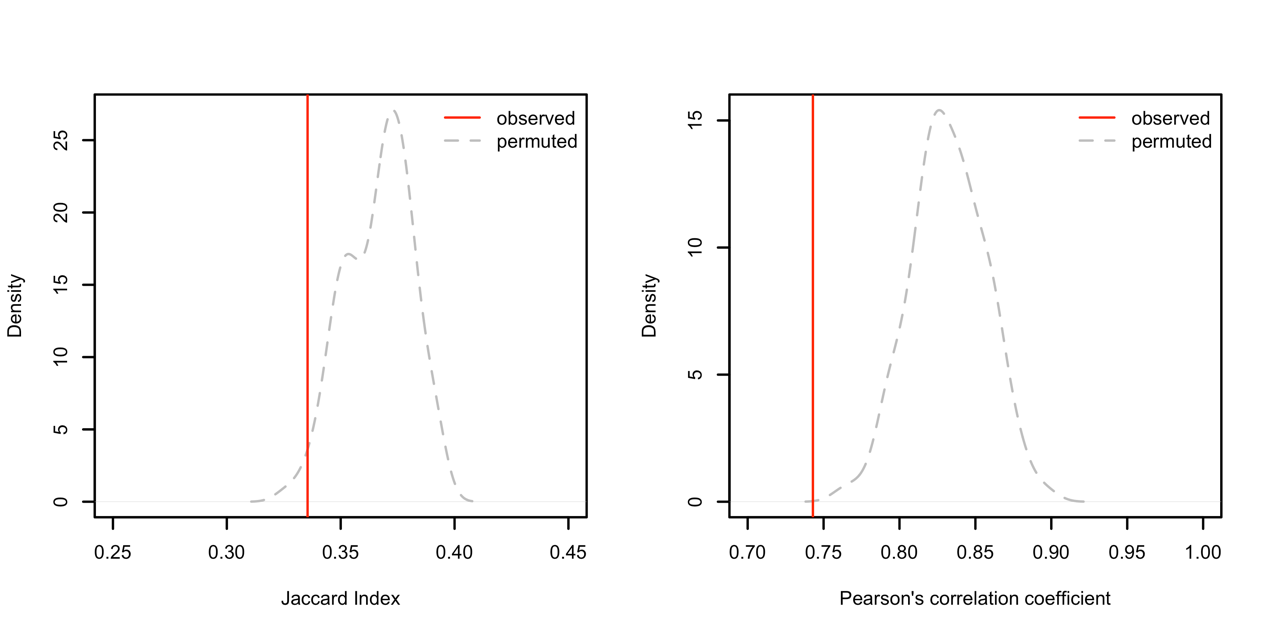
**

b

a

**Supplementary figure 2. Mean pair-wise a. Jaccard Index/ b. Pearson’s correlation coefficient for metabolome data under null expectation.** The observed mean pairwise Jaccard Index/Pearson’s correlation coefficient (red) is significantly different from the null expectation considering no population-specific evolutionary effect (grey; 100 times of permutation) (p-value = 0.02 for Jaccard Index.; p-value = 0 for r). These results indicated that the observed heterogeneity cannot be solely explained by stochasticity.

**
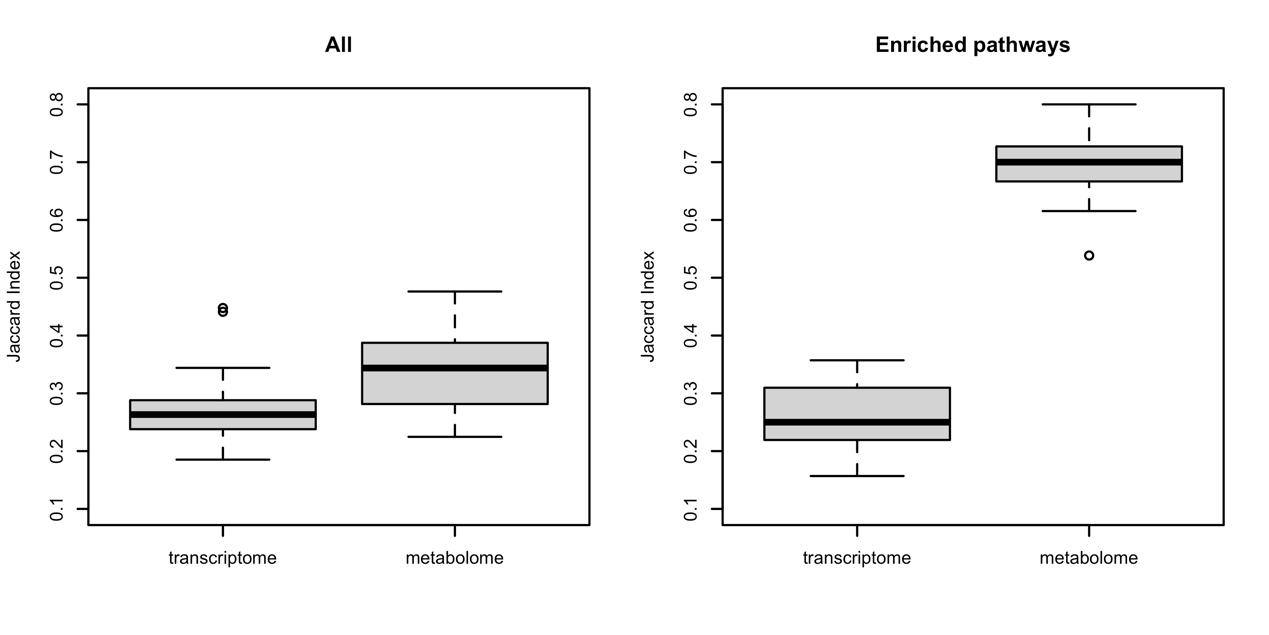
**

b

a

**Supplementary figure 3. Similarity (Jaccard Index) in the evolutionary response at gene expression and metabolite abundance across 10 populations.** **a**. The distribution of pairwise Jaccard Index among six populations (pop3, pop4, pop5, pop6, pop7, pop8) are shown. Significantly higher similarity is observed at the metabolite level (t-test, p-value < 0.001). **b**. Pairwise Jaccard Index between six populations are shown for the genes and metabolites from pathways that are enriched in all six evolved populations. Significantly higher similarity is observed at the metabolite level (t-test, n=8, p-value < 0.001).

**
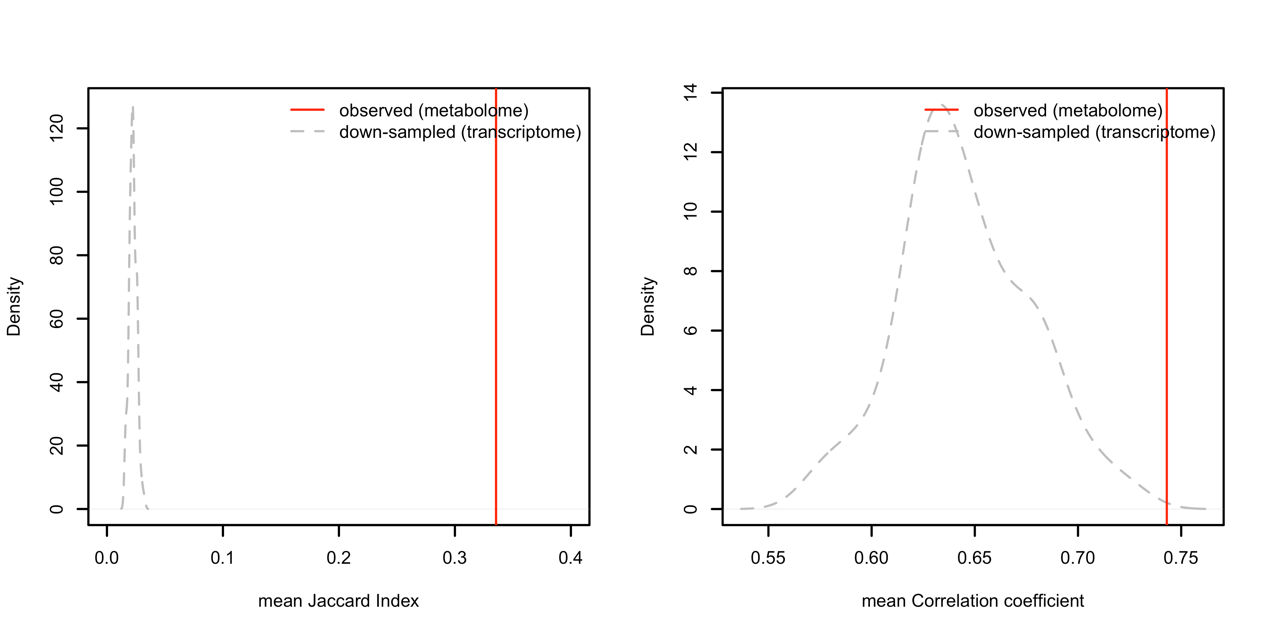
**

a

b

**Supplementary figure 4. Down sampling does not affect the difference in heterogeneity between gene expression and metabolite abundance.** **a**. The mean distribution of pairwise Jaccard Indices/Pearson’s correlation coefficients among six populations (pop3, pop4, pop5, pop6, pop7, pop8) are shown for all down sampled genes (grey, 100 times of permutation; see materials and methods) and metabolites (red). Significantly higher similarity is still observed at the metabolite level with down-sampled gene set (permutation test, p-value<0.05 for both Jaccard Index and r).

**
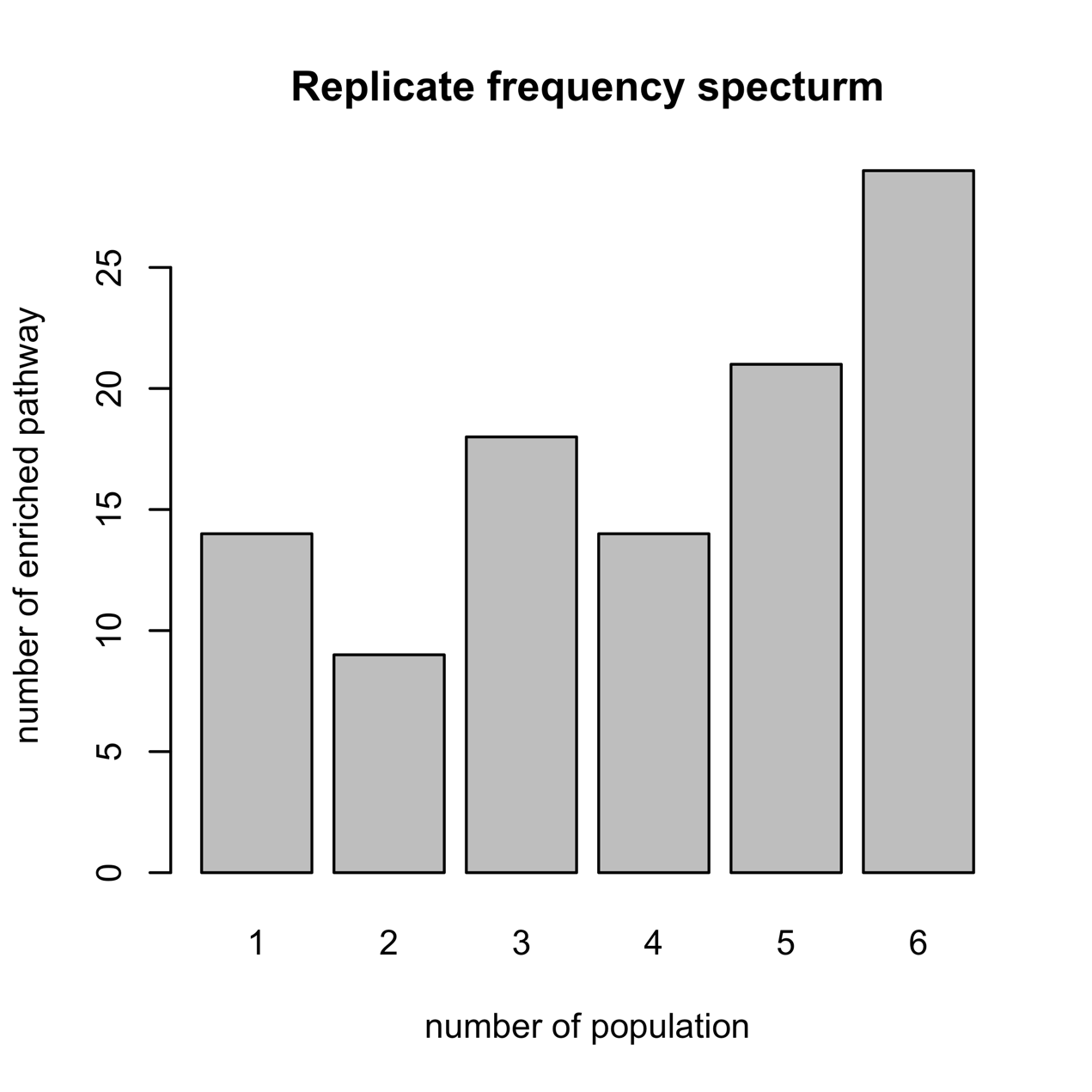
**

**Supplementary figure 5. Replicate frequency spectrum.** Number of populations (x-axis) in which a given pathway experienced a significant enrichment. The y-axis indicates the number of pathways in each category. Most of the pathways experienced a significant change in gene expression in all six evolved populations while fewer pathways were significant in few populations.

**
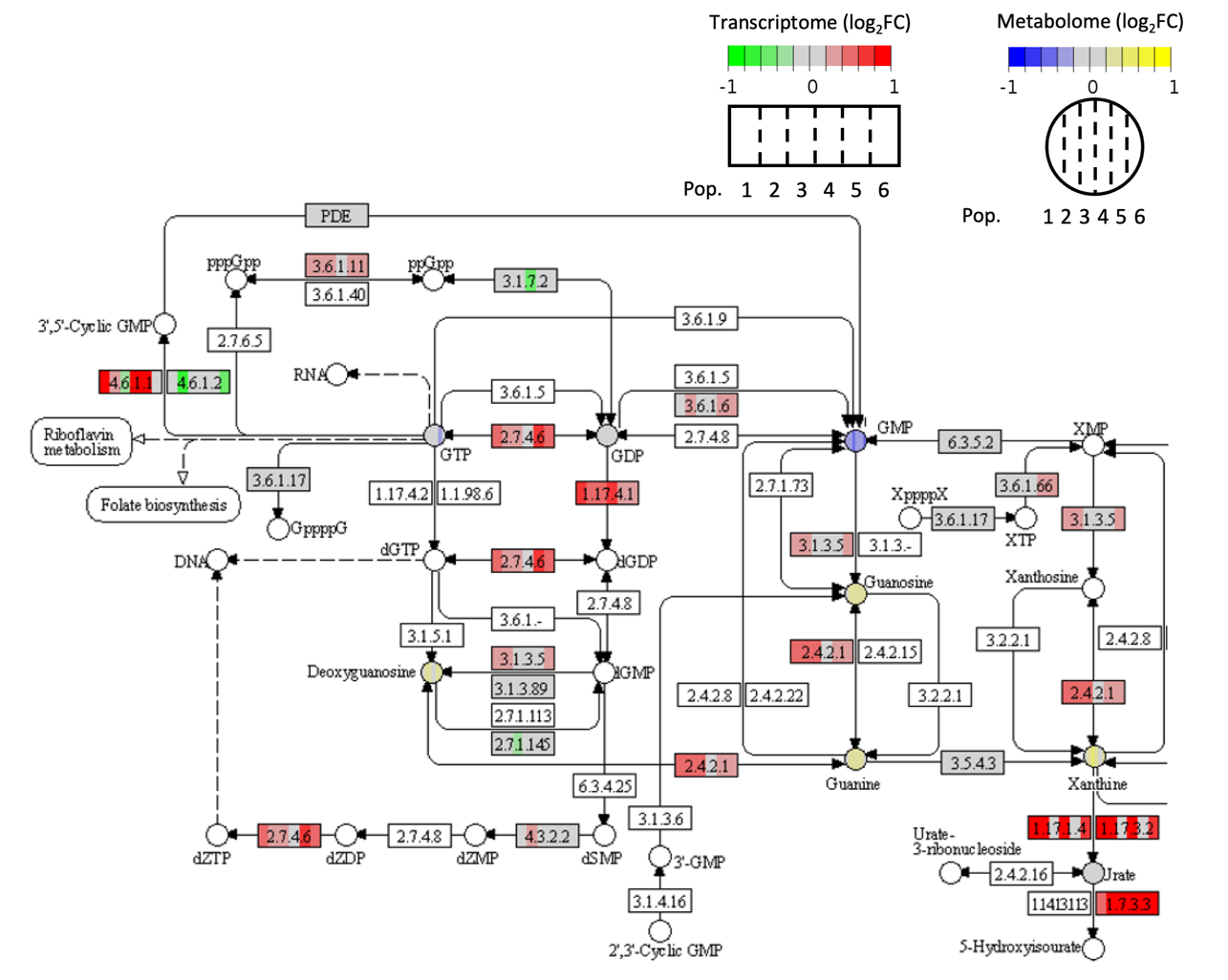
**

**Supplementary figure 6. Example of the purine metabolism KEGG pathway.** Visualization of both gene expression (rectangle) and metabolomics abundance (circle) for part of the purine metabolism pathway. Colors inside rectangle/circle indicates the evolutionary responses across evolved populations (log_2_FC, see figure legend). Each bin within each rectangle/circle indicates the evolutionary response (log_2_FC) in each evolved population (see figure legend). Grey color indicates little evolutionary response for a gene/metabolite in a certain population, while white color represents that the gene/metabolite is not detected in our data. The metabolites showed relatively more parallel changes than the protein-coding genes across evolved populations (e.g., GMP, Guanosine, Deoxyguanosine and the connected protein coding genes).

**
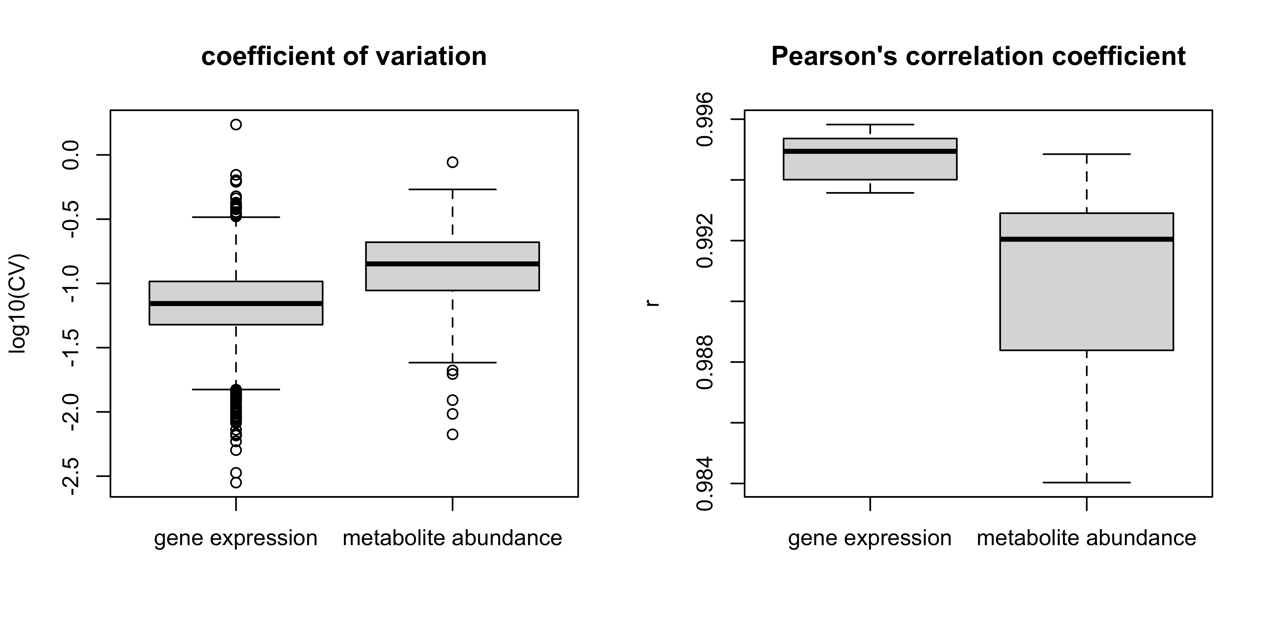
Supplementary figure 7. Variation among five ancestral population samples in transcriptomic and metabolomic data. a.** Coefficient of variation (log_10_CV; y-axis) of gene expression (CPM value) and metabolite abundance (normalized area) across five ancestral population samples. **b.** Pairwise Pearson’s correlation coefficient (r; y-axis) of gene expression (log CPM value) and metabolite abundance (log normalized area) among five ancestral population samples. Higher CV and lower correlation coefficient in metabolomic data suggests noise of the metabolomic data is larger than for the transcriptomic data.

b

a
